# Acute Kidney Injury in Urology Patients: Incidence, Causes and Outcomes

**DOI:** 10.5812/numonthly.12721

**Published:** 2013-11-13

**Authors:** Giacomo Caddeo, Simon T. Williams, Christopher W. McIntyre, Nicholas M. Selby

**Affiliations:** 1 Department of Urology, Royal Derby Hospital, Derby, UK; 2 Department of Renal Medicine, Royal Derby Hospital, Derby, UK; 3 Division of Medical Sciences and Graduate Entry Medicine, School of Medicine, University of Nottingham, Nottingham, UK

**Keywords:** Acute Kidney Injury, Urologic Diseases, Urologic Surgical Procedures, Mortality

## Abstract

**Background:**

Acute kidney injury (AKI) is common in hospitalised patients and is associated with high mortality rates. However, the epidemiology of AKI in urology patients may differ due to a higher proportion of post-renal causes and surgical procedures that result in the intentional removal of renal parenchyma.

**Objectives:**

We performed a study to examine the incidence, aetiology and outcomes of AKI in a urological population.

**Patients and Methods:**

We performed a single-centre observational study including all hospitalised patients who sustained AKI within the Urology Department over an 18 month period. Patients with AKI were prospectively identified by a hospital-wide, electronic AKI reporting system that also allows demographic, hospital admission and co-morbidity data collection. Data regarding aetiology of AKI and details of surgical procedures were added retrospectively by manual case-note search.

**Results:**

587 episodes of AKI occurred in 410 urology patients, giving an overall incidence of 6.7%. 137 (33.4%) were elective cases of whom 58 had undergone nephrectomy (radical and partial). Urinary obstruction and sepsis were the predominant causes of AKI in the 273 patients (66.6%) admitted as an emergency. Overall 30-day mortality was 7.8%; increasing severity of AKI was associated with mortality (4.8% in stage 1, 9.1% in stage 2, 14.9% in stage 3, P = 0.007). At time of discharge, only 57.7% of patients had recovered pre-morbid renal function. The observational nature of this study is a limitation, preventing determination of causality of associations.

**Conclusions:**

AKI is common in urology patients. The underlying aetiologies of AKI in this group may explain a lower overall mortality, although increasing AKI severity remains a marker of patients at higher risk of poor outcomes. The low rate of renal recovery suggests that urology patients who sustain AKI are exposed to a significant risk of CKD and its attendant consequences for long term health.

## 1. Background

Acute kidney injury (AKI), previously known as acute renal failure, is extremely common and occurs in as many as one in five hospital admissions (1). AKI is also associated with significant harm, with mortality rates above 20% and growing evidence that episodes of AKI contribute to chronic kidney disease (CKD) progression (2-4). There now exist consensus criteria that provide a method of diagnosing and describing the severity of AKI; these definitions recognise that even a small decline in renal function is associated with poor outcomes (Table 1 shows the Acute Kidney Injury Network (AKIN) criteria) (5-7). 

**Table 1.** Acute Kidney Injury Network (AKIN) Criteria for Diagnosis and Staging of Acute Kidney Injury (7)^a^

AKI Stage	Serum Creatinine Criteria	Urine Output Criteria
**1**	Increase ≥ 27 mol/L (0.3 mg/dL) within 48 hours OR Increase of 1.5-1.9 x baseline	Urine output < 0.5 mL/kg/h for > 6 hours
**2**	Increase to 2.0-2.9 x baseline	Urine output < 0.5 mL/kg/h for > 12 hours
**3**	Increase of serum creatinine to > 3 x baseline OR serum creatinine ≥ 354 mol/L (4.0 mg/dL) after a rise of at least 44 mol/L (0.5 mg/dL) OR Treatment with renal replacement therapy	Urine output < 0.3 mL/kg/h for 24 hours OR Anuria for 12 hours

^a^ During the study period the AKIN criteria were the most current, but have now been superseded the KDIGO classification that has made some minor amendments to these (7).

Urology patients are considered a high risk group for AKI due to the common occurrences of obstructive uropathy and urinary sepsis, as well as the decline in renal function that sometimes follows renal surgery. However, precise definitions of incidence and outcomes of AKI in general urology populations are lacking and although there have been some reports of the impact of specific urological procedures or conditions on renal function, current diagnostic criteria have rarely been utilised to describe these changes (8, 9). Moreover, there are coherent reasons why the epidemiology of AKI may differ in this patient group. Urinary obstruction can often be reversible by the means of surgical or interventional drainage, whereas AKI after renal surgery may not be followed by complete renal recovery as a consequence of the intentional removal of renal parenchyma. In addition, AKI following planned renal surgery may significantly differ from AKI that occurs in the setting of acute illness. 

## 2. Objectives

We performed an observational study in a generalised group of urology patients to define the incidence, aetiology and outcomes of AKI in this group.

## 3. Patients and Methods

### 3.1. Setting

The Royal Derby hospital is a 1139 bedded teaching hospital. The urology department is a tertiary referral centre for pelvic and renal surgery serving a population of approximately 700,000. There is a central chemical pathology laboratory for all inpatient and outpatient samples. A compensated kinetic Jaffe method with an inter-assay coefficient of variance of 2.3% at 96 µmol/L (Roche P-analyser, Roche Diagnostics, W. , UK) was used to measure all serum creatinine values throughout the study period.

### 3.2. Study Design

We performed an observational study to include all cases of AKI, as defined by acute changes in serum creatinine according to the AKIN criteria, within the department of urology over an 18 month period. Prospective identification of AKI cases was performed using an electronic alert system that populates a hospital wide database. From this database, all patients who had an urologist as their primary care provider as well as those who underwent a primary urological procedure were extracted. Additional clinical data regarding surgical procedures and aetiology of AKI were collected retrospectively by manual searches of case notes and electronic records.

### 3.3. Electronic Reporting System for AKI

A real-time, hospital-wide electronic reporting system based on the AKIN criteria has been developed at our centre that identifies all cases of AKI across our hospital. This system has been in clinical use since 2010 and its diagnostic accuracy has been established. A full description of the system methodology is available elsewhere (2). Following identification of a blood test from an inpatient area that is consistent with AKI, an electronic alert within the hospital’s results reporting system flags the presence of AKI to the responsible clinician and indicates severity by reporting the AKI stage. This system also allows prospective data collection for all cases of AKI at our centre. These data are supplemented by highest AKI stage, last serum creatinine in stay (to assess renal recovery), length of hospital stay and patient survival (at discharge and 30 days). Approval to use routinely collected anonymised data in this way has been obtained from the National In formation Governance Board. 

### 3.4. Statistical Analysis

Parametric data are presented as mean ± standard deviation and non-parametric data as median (inter-quartile range). Chi-squared test was used to compare categorical data and t-test or Mann-Whitney test to compare continuous data depending on whether data were parametric or non-parametric. P-values of < 0.05 were considered significant. All analyses were performed using SPSS v19 (IBM Corp).

## 4. Results

From August 2010 until February 2012, a total of 587 episodes of AKI occurred in 410 urology patients. This corresponded to an incidence of 6.7% in the total hospitalised urology population. The male : female ratio was 4:1 and mean age was 73 ± 13 years. 61.0% of affected patients had AKI stage 1, 16.1% had stage 2 and 22.9% had stage 3. The majority of the patients (88.7%) sustained a single AKI episode whereas 11.3% had more than one AKI, ranging from two to four episodes per patient. Two-thirds of the patients (273 cases, 66.6%) were admitted to the hospital as an emergency, whilst half that number (137 cases, 33.4%) sustained AKI during the course of an elective admission. Median length of stay was 7 days (IQR 8) for elective admissions and 7 days (IQR 11) for emergency admissions.

### 4.1. AKI in Elective Admissions

Data regarding the procedures performed in the elective group were available for 111 out of 137 cases (81%). The procedure most frequently associated with AKI was nephrectomy (radical and partial) and nephroureterectomy (58 pts, 42.3%). Of these patients 52 had AKI stage 1 (89.7%), three had AKI stage 2 (5.2%) and two had AKI stage 3 (3.4%). No deaths occurred in these patients. AKI was observed following TURBT in 21 pts, accounting for 15.3% of the elective group, with a pre-renal mechanism in nine (post-operative bleeding or sepsis) and a post-renal cause in six (ureteric orifice obstruction). In the remaining six cases, it was not possible to determine the aetiology of AKI. In the third largest category, 10 patients (5.8%) sustained AKI after radical cystectomy. In seven of these patients AKI was related to a pre-renal cause (haemorrhage, sepsis, cardiovascular complications) whereas in three the aetiology was primarily obstructive (accidental removal of ureteric stent, anastomotic leak, dislodgement of pre-existing percutaneous nephrostomy). The remaining procedures complicated by post-operative AKI are displayed in Table 2 and include open and endoscopic surgery of both upper and lower urinary tracts. In the majority of these cases, it was possible to identify urinary obstruction, sepsis or a combination of both as the likely aetiology of AKI.

**Table 2.** Frequency Describing the Elective Urological Procedures that Were Complicated by AKI

Procedures	No.	%
**Nephrectomy (radical + partial) + nephroureterectomy**	59	43.1
**TURBT** ^**a**^	21	15.3
**Cystoprostatectomy**	8	5.8
**Ureteroscopic lithotripsy**	5	3.6
**TURP** ^**a**^	3	2.2
**Radical prostatectomy**	2	1.5
**JJ stent insertion**	2	1.5
**Ileal conduit formation**	1	0.7
**Anterior exenteration**	1	0.7
**JJ stent removal**	1	0.7
**Segmental ureterectomy**	1	0.7
**Endoscopic ureteric biopsy**	1	0.7
**Total penectomy**	1	0.7
**Total pelvic exenteration**	1	0.7
**Nephrostomy removal**	1	0.7
**Flexible cystoscopy**	1	0.7
**ESWL** ^**a**^	1	0.7
**PCNL** ^**a**^	1	0.7
**Other-Not known**	26	18.2
**Total**	137	100.0

^a^ Abbreviations: ESWL, extracorporeal shockwave lithotripsy; PCNL, percutaneous nephrolithotomy; TURBT, transurethral resection of bladder tumour; TURP, transurethral resection of prostate.

### 4.2. AKI in Non-elective Admissions

In the 273 patients with AKI who were admitted as an emergency the three most frequent primary diagnoses were urinary retention (58 pts, 21.2%), urinary tract sepsis/peno-scrotal infections (39 pts, 14.3%) and obstructing urinary calculi (34 pts, 12.5%). A full list of conditions associated with AKI in this group is included in Table 3. A comparison between the characteristics of patients in the elective and non-elective admission groups is shown in Table 4, showing that the non-elective group were older, had a higher proportion of males as well as a greater proportion of patients with more severe AKI.

**Table 3.** Frequency Table Describing the Primary Diagnosis in Non-elective Urological Admissions that were Complicated by AKI

Primary Diagnoses	No.	%
**Retention of urine - benign cause**	58	21.2
**UTI, peno-scrotal infections, sepsis**	41	15.0
**Obstructing ureteral/renal calculi **	34	12.5
**Bladder Cancer**	27	9.9
Hydronephrosis	24	8.8
Hematuria/clot retention	3	1.1
**Hematuria**	17	6.2
Catheter related	9	
BPH related	4	
Hematological causes	1	
Nephrostomy related	1	
Suprapubic catheter related	1	
Nephrological causes	1	
**Non-lithiasic benign hydronephrosis (PUJO, ureteral strictures/compression)**	15	5.5
**Prostate Cancer**	11	4.0
Urinary retention	4	
Metastatic, end stage disease	4	
Ureteric obstruction/hydronephrosis	2	
Sepsis and hematuria post prostatic biopsy	1	
**Mechanical complications of genitourinary stents/malfunction of urostomy**	14	5.1
**Other Cancer**	4	1.5
Transitional cell carcinoma of upper tract	2	
Malignant colovesical fistula in locally advanced carcinoma of anus	1	
Bladder invasion from caecal carcinoma	1	
**Renal Cancer**	4	1.5
Hematuria, locally advanced disease	3	1
Metastatic disease	1	1
Other not specified	50	18.3
**Total**	273	100.0

^a^Abbreviations: BPH, benign prostatic hypertrophy; PUJO, pelvi-ureteric junction obstruction; UTI, urinary tract infection.

**Table 4.** Comparison of Elective and Emergency Urology Patients With AKI^a^

Admission Type	Elective	Emergency	P value
**Number of patients, No. (%)**	137 (33.4)	273 (66.6)	-
**Age, Mean (SD), y**	70.7 (11)	74.5 (14)	0.03
**Male patients **	70.8%	84.6%	0.001
**AKI stage, No. (%)**			0.007^a^
1	98 (71.5)	152 (55.7)	
2	18 (13.1)	48 (17.6)	
3	21 (15.3)	73 (26.7)	
**30-day mortality, No. (%)**	5 (3.6)	27 (9.9)	0.031
**Renal recovery at discharge **	44.5%	64.5%	< 0.001
**Median length of stay (IQR)**	7 (8)	7 (11)	0.73

^a^chi square for trend.

### 4.3. Patient Outcomes

Overall, the 30-day mortality rate in urological patients with AKI was 7.8% (32 patients). The majority of deaths occurred in non-elective patients (27 pts, mortality rate 9.9%); five deaths occurred in the elective admission group (mortality rate 3.6%, comparison versus non-elective group P = 0.013). In the latter group, the cause of death in one patient was sepsis following complicated flexible ureteroscopy and laser lithotripsy of a renal stone, whilst the remaining four patients had progression of previously diagnosed advanced urological malignancy (two prostate cancers and two bladder cancers).

**Figure 1.**
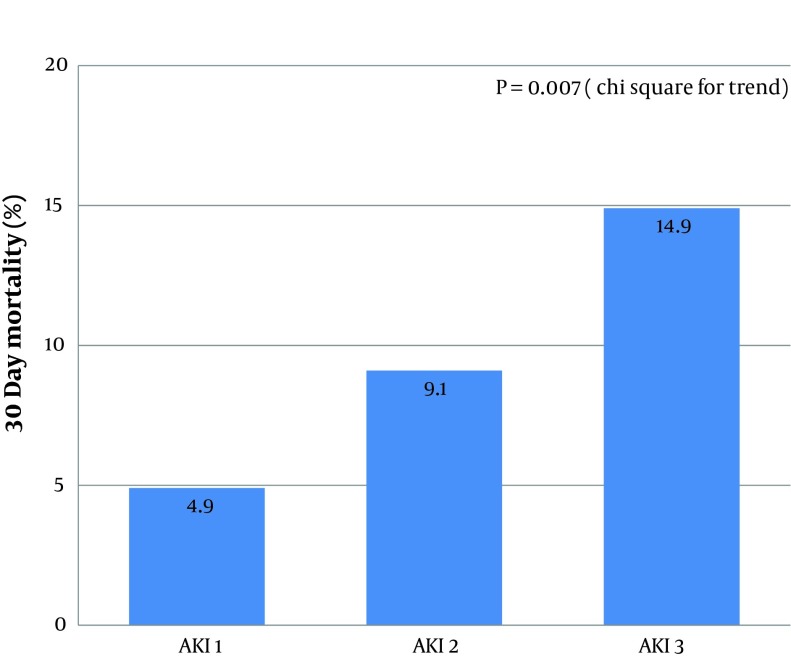
30 Day Mortality Stratified by AKI Stage

Factors associated with mortality were examined. The severity of AKI was important, with increasing mortality rates seen with more severe AKI. Mortality was 4.8% in stage 1 AKI, 9.1% in stage 2 AKI and 14.9% in stage 3 AKI (chi square for trend P = 0.007). These data are summarised in Figure 1. As expected, patients who died within 30 days were older as compared to those who survived (mean age 78.8 +/- 11.5 years versus 72.7 +/- 13.5 years, P = 0.013). In those with CKD, 30-day mortality rates were 13% as compared to 5.4% in those with normal baseline renal function (P = 0.019). 22 of the non-survivors (68.8%) had a known malignancy and 20 of those had metastatic disease. The 30-day mortality in the cancer group was 12.9% versus 4.2% in those without cancer (P = 0.001) and in those with metastatic disease 47.1% as compared to 2.2%, P < 0.001. 

At time of discharge, complete renal recovery (defined as a creatinine less than 27 mol/L above baseline) occurred in only 237 patients (57.7%). Non-elective patients were more likely to achieve complete renal recovery as compared to the elective admission group (64.5% versus 44.5%, P < 0.001). Within the group of 58 nephrectomies (all types) renal recovery was observed only in 23 patients (39.7%). Numbers were too small for statistical comparisons, but four of seven patients (57%) undergoing partial nephrectomy achieved complete renal recovery as compared with 19 from 51 (37%) in those undergoing radical nephrectomy.

## 5. Discussion

This study provides the first description of the epidemiology of AKI in a urology population, using current diagnostic criteria and including all subgroups of patients. The results confirm that AKI is common in this population and its severity remains associated with mortality, although this appears lower than in other groups. However, the rate of renal recovery was low.

The widespread acceptance of the RIFLE, AKIN and more recently the KDIGO definitions for AKI have emphasised its high incidence and extremely poor outcomes (5-7).

In part, a better appreciation of its high incidence stems from the recognition that even small declines in renal function are associated with increased mortality, longer hospital stays and increased healthcare costs (10). Using current definitions, the reported incidence of AKI in general hospitalised patients varies between five and twenty percent of all admissions with overall mortality rates above 20% (1, 2). Mortality rates increase with severity of AKI being greater than 30% in stage 3 AKI, rising to more than 45% in critical care patients requiring renal replacement therapy (3). AKI rarely occurs in isolation and co-existing acute illness is often implicated in its aetiology as well as having a strong impact on patient outcome (11). However, AKI is more than a simple marker of illness severity; it is increasingly appreciated that AKI has distant effects that contribute to organ dysfunction and negatively impact on overall outcomes (12).

Although urology patients are generally accepted to be a high risk population for AKI, no previous studies have described its epidemiology in this group. Some reports describe changes in renal function in relation to a specific urological condition but few have employed current diagnostic criteria. One study used a modification of the AKIN criteria to describe the features of AKI secondary to ureteral calculi, but the authors did not report the severity of AKI or examine its effect on outcomes (8). In another example, AKI in bilateral ureteric obstruction was defined as ≥ 33% decrease in serum creatinine after intervention (9). The use of such alternative descriptions makes comparisons between studies very difficult.

Our results confirm that AKI is a common clinical problem facing urologists although within this population there are several distinct groups. In those undergoing elective surgery, almost half of the patients classified as AKI had undergone partial or radical nephrectomy. In these patients, the more severe stages of AKI were rare with no mortality. This good early prognosis is obviously at odds with the outcomes of AKI in other clinical situations and highlights that AKI is not a diagnosis in itself but a syndrome describing changes in renal function. Its implications therefore depend on the underlying aetiology and co-existing acute conditions (11). Although ‘apparent AKI’ in stable patients undergoing nephrectomy may sometimes occur as a result of nephron loss, post-operative patients may develop AKI because of other complications, which in our series was largely due to obstructive causes or sepsis. The other two elective procedures in which AKI was observed with greatest frequency were TURBT and cystectomy. As with any major surgery, the latter is recognised to have a significant complication rate (13) but it is not possible to determine from our results whether AKI associated with TURBT reflects the larger number of cases performed or whether there is an increased risk of AKI specific to this procedure.

Overall mortality rates were lower than seen in studies of other AKI populations (1, 2, 14). This may reflect a greater proportion of elective patients (which included those undergoing nephrectomy), which is in contrast to general hospitalised AKI patients in whom over 90% are admitted as an emergency and AKI occurs as part of acute illness (15). In addition, urinary obstruction was a common underlying cause in which AKI may be more easily reversible by radiological or surgical drainage (9). Despite this, it is important to note that mortality progressively increased with AKI stage. The presence and severity of AKI remains a marker of the ‘unwell patient’ who requires additional clinical attention.

Two co-morbid conditions had appeared to have an influence on outcomes. Firstly, we observed an association between CKD and mortality, which is particularly important as CKD is a strong risk factor for developing AKI in this first instance. Secondly, there was also a strong link between malignancy and mortality, which was present in two-thirds of non-survivors and accounted for all but one of the deaths in the elective group. These results are consistent with other reports in which a malignant cause of obstructive uropathy conferred a significantly worse prognosis (9). In contrast to historical data, more recent studies in general AKI populations suggest that malignancy is now the third most common cause of death (15). In part, this may reflect that a significant proportion of patients are admitted to hospital to receive end of life care (16) and in some AKI may occur as part of the terminal phase of their illness.

The low rate of renal recovery by time of hospital discharge may in part reflect the inclusion of nephrectomy patients. It is important to note that AKI occurring after partial nephrectomy increases the likelihood of developing subsequent CKD (17), which in turn contributes to increased cardiovascular risk (18-20). However, renal surgery was not the only explanation for non-recovery by time of hospital discharge. This observation is consistent with the burgeoning evidence that links episodes of AKI to the onset and progression of CKD. Although our follow up time was short, it would appear that this paradigm may also apply to urological patients (4).

There are some weaknesses to our study, in particular its observational nature precludes the determination of causality of the reported associations. Patients’ co-morbidity was derived from hospital coding data which may introduce some inaccuracies.

Finally, our methodology prevented the inclusion of a control group without AKI. We are also aware that the cohort of patients is probably too heterogeneous to allow for definite conclusions from simple parametric and non-parametric statistical testing. However, although this was a heterogeneous group, and although the conclusions are to be expected, these have not previously been described and they contribute to raise awareness of AKI in Urology.

Conclusion: This study is the first to describe incidence, aetiology and outcome of AKI occurring in elective and emergency urology admissions. It is clear that the underlying aetiology of AKI must be taken into consideration when assessing the potential impact of AKI but that the presence of AKI remains a marker of the unwell patient. Specific strategies to target improvements in renal recovery following AKI may be particularly pertinent in this patient group.
